# 10-Benzoyl­phenanthrene-8,9-dicarboxylic anhydride

**DOI:** 10.1107/S1600536809052684

**Published:** 2009-12-12

**Authors:** Hai-Tao Yu, Yi Wei, Yan Zhang

**Affiliations:** a School of Chemistry and Chemical Engineering, Nanjing University, Nanjing 210093, People’s Republic of China

## Abstract

The asymmetric unit of the title compound, C_23_H_12_O_4_, contains two nearly parallel independent mol­ecules; the dihedral angles between the phenanthrene ring systems of the two mol­ecules and between the benzene rings of the two mol­ecules are 4.97 (9) and 8.1 (2)°, respectively. In each mol­ecule, the benzene ring is nearly perpendicular to the phenanthrene ring system, with dihedral angles of 86.42 (19) and 86.68 (18)°. π–π stacking exists between the phenanthrene ring systems of the two independent mol­ecules [centroid–centroid distance = 3.698 (2) Å]. Short intermolec­ular contacts [O⋯O = 2.86 (2) and C⋯O = 2.88 (2) Å] are also present in the crystal structure.

## Related literature

The title compound is an important inter­mediate for the synthesis of azonafide [systematic name 2-[2′-(dimethyl-amino)ethyl]-1,2-dihydro-3*H*-dibenz[*de*,*h*]isoquinoline-1,3-dione] derivatives; for the anti­tumor properties of azonafide and its analogues, see: Sami *et al.* (2000 ▶); Hutchings *et al.* (1988 ▶). For the synthesis, see: Zhang *et al.* (2000 ▶).
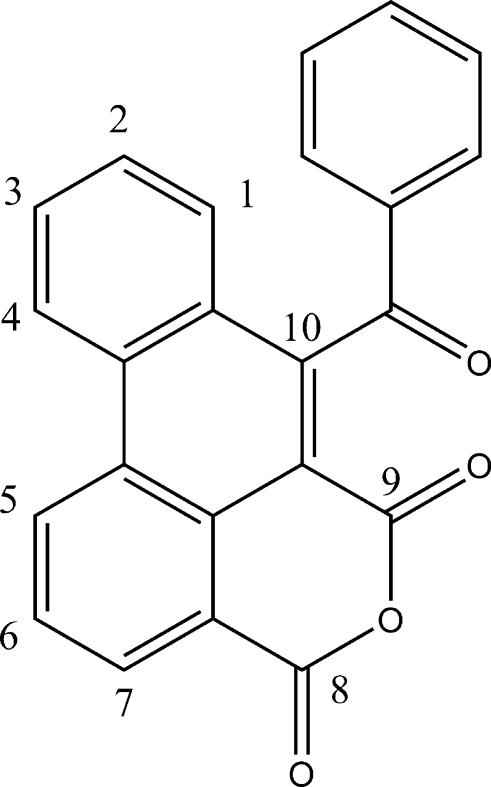


## Experimental

### 

#### Crystal data

C_23_H_12_O_4_
*M* *_r_* = 352.33
Monoclinic, 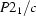

*a* = 11.329 (2) Å
*b* = 17.767 (4) Å
*c* = 16.811 (3) Å
β = 99.64 (3)°
*V* = 3336.0 (11) Å^3^
*Z* = 8
Mo *K*α radiation
μ = 0.10 mm^−1^
*T* = 293 K
0.30 × 0.28 × 0.26 mm

#### Data collection

Enraf–Nonius CAD-4 diffractometer
6073 measured reflections
6073 independent reflections
2895 reflections with *I* > 2σ(*I*)
3 standard reflections every 3 min
intensity decay: 1%

#### Refinement

*R*[*F* ^2^ > 2σ(*F* ^2^)] = 0.066
*wR*(*F* ^2^) = 0.160
*S* = 1.01
6073 reflections
487 parameters
H-atom parameters constrained
Δρ_max_ = 0.17 e Å^−3^
Δρ_min_ = −0.17 e Å^−3^

### 

Data collection: *CAD-4 EXPRESS* (Enraf–Nonius, 1994 ▶); cell refinement: *CAD-4 EXPRESS*; data reduction: *XCAD4* (Harms & Wocadlo, 1995 ▶); program(s) used to solve structure: *SHELXS97* (Sheldrick, 2008 ▶); program(s) used to refine structure: *SHELXL97* (Sheldrick, 2008 ▶); molecular graphics: *ORTEP-3 for Windows* (Farrugia, 1997 ▶); software used to prepare material for publication: *SHELXL97*.

## Supplementary Material

Crystal structure: contains datablocks I, global. DOI: 10.1107/S1600536809052684/xu2667sup1.cif

Structure factors: contains datablocks I. DOI: 10.1107/S1600536809052684/xu2667Isup2.hkl

Additional supplementary materials:  crystallographic information; 3D view; checkCIF report

## Figures and Tables

**Fig. 1.**
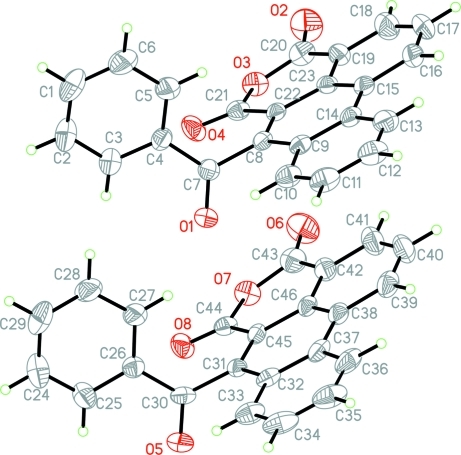
The molecular structure of the title compound with displacement ellipsoids shown at 30% probability ellipsoids for non-H atoms.

## supplementary crystallographic information

### Comment

Previous articles have described the preparation and antitumor properties of azonafide and many analogues with structural variations in the side chain and the bent phenanthrene nucleus (Sami, 2000; Hutchings, 1988). In this paper, we present the X-ray crystallographic analysis of the title compound (Fig. 1), which is an important intermediate for the synthesis of azonafide derivatives. The centroids distance between nearly parallel C10-benzene and C40-benzene rings [dihedral angle 4.99°] is 3.698 (2) Å, which suggests the existence of π-π stacking in the crystal structure. Intermolecular sorter contacts [O···O 2.86 Å and C···O 2.88 Å] are present in the crystal structure.

### Experimental

A solution of homophthalic anhydride (176 mg, 1 mmol) and diphenyl acetylene (356 mg, 2 mmol) in anhydrous acetonitrile (50 ml) was purged with dry argon for 10 min and then irradiated for 48 h under continuous argon purging. The single crystals of the title compound were obtained from the reaction mixture. The light source was a medium-pressure mercury lamp (500 W) in a cooling water jacket that was further surrounded by a layer of filter solution (1 cm thick, 20% aqueous sodium nitrite) to cut off light of wavelength shorter than 400 nm (Zhang *et al.*,  2000).

### Refinement

H atoms were positioned geometrically and refined using a riding model, with C—H = 0.93 Å and *U*_iso_(H) = 1.2*U*_eq_(C).

### Crystal data

**Table:**

C_23_H_12_O_4_	*F*(000) = 1456
*M**_r_* = 352.33	*D*_x_ = 1.403 Mg m^−^^3^
Monoclinic, *P*2_1_/*c*	Mo *K*α radiation, λ = 0.71073 Å
Hall symbol: -P 2ybc	Cell parameters from 25 reflections
*a* = 11.329 (2) Å	θ = 10.8–15.2°
*b* = 17.767 (4) Å	µ = 0.10 mm^−^^1^
*c* = 16.811 (3) Å	*T* = 293 K
β = 99.64 (3)°	Block, colourless
*V* = 3336.0 (11) Å^3^	0.30 × 0.28 × 0.26 mm
*Z* = 8	

### Data collection

**Table:**

Enraf–Nonius CAD-4 diffractometer	*R*_int_ = 0.0000
Radiation source: fine-focus sealed tube	θ_max_ = 25.3°, θ_min_ = 1.7°
graphite	*h* = −13→13
ω/2θ scans	*k* = 0→21
6073 measured reflections	*l* = 0→20
6073 independent reflections	3 standard reflections every 120 min
2895 reflections with *I* > 2σ(*I*)	intensity decay: 1%

### Refinement

**Table:**

Refinement on *F*^2^	Primary atom site location: structure-invariant direct methods
Least-squares matrix: full	Secondary atom site location: difference Fourier map
*R*[*F*^2^ > 2σ(*F*^2^)] = 0.066	Hydrogen site location: inferred from neighbouring sites
*wR*(*F*^2^) = 0.160	H-atom parameters constrained
*S* = 1.01	*w* = 1/[σ^2^(*F*_o_^2^) + (0.0558*P*)^2^] where *P* = (*F*_o_^2^ + 2*F*_c_^2^)/3
6073 reflections	(Δ/σ)_max_ < 0.001
487 parameters	Δρ_max_ = 0.17 e Å^−^^3^
0 restraints	Δρ_min_ = −0.17 e Å^−^^3^

### Special details

**Table:**

Geometry. All e.s.d.'s (except the e.s.d. in the dihedral angle between two l.s. planes) are estimated using the full covariance matrix. The cell e.s.d.'s are taken into account individually in the estimation of e.s.d.'s in distances, angles and torsion angles; correlations between e.s.d.'s in cell parameters are only used when they are defined by crystal symmetry. An approximate (isotropic) treatment of cell e.s.d.'s is used for estimating e.s.d.'s involving l.s. planes.
Refinement. Refinement of *F*^2^ against ALL reflections. The weighted *R*-factor *wR* and goodness of fit *S* are based on *F*^2^, conventional *R*-factors *R* are based on *F*, with *F* set to zero for negative *F*^2^. The threshold expression of *F*^2^ > σ(*F*^2^) is used only for calculating *R*-factors(gt) *etc*. and is not relevant to the choice of reflections for refinement. *R*-factors based on *F*^2^ are statistically about twice as large as those based on *F*, and *R*- factors based on ALL data will be even larger.

### Fractional atomic coordinates and isotropic or equivalent isotropic displacement parameters (Å^2^)

**Table:**

	*x*	*y*	*z*	*U*_iso_*/*U*_eq_	
C1	0.2673 (5)	0.1589 (3)	−0.0305 (3)	0.0998 (16)	
H1A	0.2295	0.1738	−0.0814	0.120*	
C2	0.3718 (5)	0.1933 (2)	0.0036 (3)	0.0840 (13)	
H2A	0.4037	0.2320	−0.0234	0.101*	
C3	0.4290 (3)	0.1700 (2)	0.0780 (2)	0.0644 (11)	
H3A	0.5007	0.1925	0.1010	0.077*	
C4	0.3807 (3)	0.11345 (19)	0.1192 (2)	0.0524 (9)	
C5	0.2739 (3)	0.0805 (3)	0.0847 (3)	0.0842 (14)	
H5A	0.2398	0.0430	0.1122	0.101*	
C6	0.2179 (4)	0.1032 (3)	0.0093 (3)	0.1071 (17)	
H6A	0.1466	0.0806	−0.0145	0.129*	
C7	0.4459 (3)	0.08948 (18)	0.1994 (2)	0.0481 (9)	
C8	0.3825 (3)	0.03796 (17)	0.24980 (19)	0.0411 (8)	
C9	0.3994 (3)	−0.04183 (18)	0.2433 (2)	0.0444 (8)	
C10	0.4757 (3)	−0.0711 (2)	0.1935 (2)	0.0604 (10)	
H10A	0.5160	−0.0386	0.1640	0.072*	
C11	0.4915 (3)	−0.1475 (2)	0.1880 (2)	0.0717 (12)	
H11A	0.5429	−0.1663	0.1551	0.086*	
C12	0.4326 (4)	−0.1959 (2)	0.2302 (3)	0.0743 (12)	
H12A	0.4444	−0.2475	0.2260	0.089*	
C13	0.3567 (3)	−0.1698 (2)	0.2786 (2)	0.0667 (11)	
H13A	0.3158	−0.2039	0.3059	0.080*	
C14	0.3385 (3)	−0.09211 (18)	0.2883 (2)	0.0485 (9)	
C15	0.2630 (3)	−0.06240 (19)	0.3414 (2)	0.0474 (9)	
C16	0.1970 (3)	−0.1080 (2)	0.3880 (2)	0.0666 (11)	
H16A	0.2024	−0.1601	0.3849	0.080*	
C17	0.1263 (4)	−0.0769 (3)	0.4367 (3)	0.0800 (13)	
H17A	0.0834	−0.1080	0.4659	0.096*	
C18	0.1174 (4)	0.0001 (3)	0.4437 (3)	0.0812 (13)	
H18A	0.0664	0.0249	0.4754	0.097*	
C19	0.1796 (3)	0.0465 (2)	0.4003 (2)	0.0540 (9)	
C22	0.3123 (3)	0.06531 (17)	0.30131 (19)	0.0406 (8)	
C23	0.2525 (3)	0.01599 (18)	0.34878 (19)	0.0427 (8)	
O6	0.5567 (3)	0.11817 (18)	0.44109 (18)	0.0940 (10)	
O7	0.7026 (2)	0.15182 (13)	0.37616 (14)	0.0592 (7)	
C24	0.8948 (5)	0.2090 (2)	0.0165 (3)	0.0858 (14)	
H24A	0.9355	0.2427	−0.0115	0.103*	
C25	0.9474 (4)	0.1832 (2)	0.0912 (2)	0.0680 (11)	
H25A	1.0241	0.1990	0.1134	0.082*	
C26	0.8861 (3)	0.13347 (19)	0.1335 (2)	0.0515 (9)	
C27	0.7738 (3)	0.1094 (2)	0.0989 (2)	0.0773 (13)	
H27A	0.7327	0.0754	0.1262	0.093*	
C28	0.7218 (4)	0.1355 (3)	0.0239 (3)	0.0956 (16)	
H28A	0.6456	0.1192	0.0012	0.115*	
C29	0.7815 (5)	0.1847 (3)	−0.0169 (3)	0.0898 (15)	
H29A	0.7459	0.2021	−0.0674	0.108*	
C31	0.8870 (3)	0.04461 (17)	0.25475 (19)	0.0419 (8)	
C32	0.9170 (3)	−0.03219 (18)	0.2396 (2)	0.0476 (9)	
C33	1.0017 (3)	−0.0493 (2)	0.1906 (2)	0.0663 (11)	
H33A	1.0385	−0.0106	0.1667	0.080*	
C34	1.0310 (4)	−0.1227 (3)	0.1774 (3)	0.0824 (14)	
H34A	1.0885	−0.1335	0.1455	0.099*	
C35	0.9752 (4)	−0.1806 (2)	0.2114 (3)	0.0856 (15)	
H35A	0.9952	−0.2302	0.2019	0.103*	
C36	0.8920 (4)	−0.1663 (2)	0.2583 (3)	0.0731 (12)	
H36A	0.8549	−0.2063	0.2800	0.088*	
C37	0.8601 (3)	−0.09157 (18)	0.2752 (2)	0.0557 (10)	
C38	0.7729 (3)	−0.0747 (2)	0.3260 (2)	0.0539 (9)	
C39	0.7101 (4)	−0.1307 (2)	0.3626 (2)	0.0723 (12)	
H39A	0.7264	−0.1813	0.3551	0.087*	
C40	0.6263 (4)	−0.1115 (3)	0.4086 (3)	0.0822 (13)	
H40A	0.5859	−0.1491	0.4317	0.099*	
C41	0.6012 (4)	−0.0382 (3)	0.4211 (2)	0.0766 (12)	
H41A	0.5427	−0.0261	0.4518	0.092*	
C42	0.6612 (3)	0.0183 (2)	0.3890 (2)	0.0555 (9)	
C43	0.6329 (4)	0.0960 (3)	0.4042 (2)	0.0675 (11)	
C45	0.8067 (3)	0.05975 (17)	0.30482 (19)	0.0408 (8)	
C46	0.7477 (3)	0.00066 (18)	0.34088 (19)	0.0447 (8)	
O8	0.8217 (2)	0.19227 (13)	0.29575 (16)	0.0649 (7)	
O1	0.54992 (19)	0.10725 (13)	0.22378 (14)	0.0591 (7)	
C44	0.7792 (3)	0.1380 (2)	0.3224 (2)	0.0483 (9)	
O3	0.2359 (2)	0.17298 (12)	0.36796 (15)	0.0591 (7)	
O4	0.3390 (2)	0.19411 (13)	0.27183 (17)	0.0680 (8)	
O5	1.0484 (2)	0.12609 (14)	0.24210 (15)	0.0681 (7)	
O2	0.1008 (3)	0.15884 (17)	0.44693 (18)	0.0973 (10)	
C21	0.2981 (3)	0.14706 (19)	0.3096 (2)	0.0476 (9)	
C30	0.9469 (3)	0.10541 (19)	0.2136 (2)	0.0509 (9)	
C20	0.1671 (4)	0.1276 (2)	0.4076 (2)	0.0634 (10)	

### Atomic displacement parameters (Å^2^)

**Table:**

	*U*^11^	*U*^22^	*U*^33^	*U*^12^	*U*^13^	*U*^23^
C1	0.117 (4)	0.113 (4)	0.059 (3)	0.017 (4)	−0.016 (3)	0.021 (3)
C2	0.123 (4)	0.065 (3)	0.061 (3)	0.003 (3)	0.009 (3)	0.016 (2)
C3	0.077 (3)	0.059 (3)	0.057 (3)	0.002 (2)	0.010 (2)	0.006 (2)
C4	0.052 (2)	0.056 (2)	0.047 (2)	0.0066 (19)	0.0024 (18)	0.0072 (18)
C5	0.057 (3)	0.117 (4)	0.072 (3)	−0.018 (3)	−0.005 (2)	0.027 (3)
C6	0.070 (3)	0.162 (5)	0.078 (4)	−0.008 (3)	−0.021 (3)	0.034 (4)
C7	0.049 (2)	0.041 (2)	0.055 (2)	0.0016 (17)	0.0100 (19)	−0.0010 (17)
C8	0.0389 (18)	0.0389 (19)	0.043 (2)	0.0027 (15)	−0.0009 (16)	0.0013 (16)
C9	0.0410 (19)	0.042 (2)	0.048 (2)	0.0076 (16)	−0.0002 (17)	−0.0055 (17)
C10	0.055 (2)	0.056 (2)	0.068 (3)	0.0075 (19)	0.005 (2)	−0.008 (2)
C11	0.065 (3)	0.068 (3)	0.080 (3)	0.018 (2)	0.006 (2)	−0.020 (2)
C12	0.065 (3)	0.046 (2)	0.105 (4)	0.005 (2)	−0.007 (3)	−0.018 (2)
C13	0.056 (2)	0.044 (2)	0.095 (3)	−0.0048 (19)	−0.003 (2)	0.000 (2)
C14	0.045 (2)	0.0349 (19)	0.060 (2)	0.0001 (16)	−0.0083 (18)	0.0005 (17)
C15	0.042 (2)	0.047 (2)	0.049 (2)	−0.0037 (17)	−0.0030 (17)	0.0033 (17)
C16	0.060 (3)	0.059 (3)	0.077 (3)	−0.010 (2)	−0.001 (2)	0.019 (2)
C17	0.065 (3)	0.090 (4)	0.091 (3)	−0.008 (3)	0.030 (3)	0.029 (3)
C18	0.076 (3)	0.084 (3)	0.091 (3)	0.006 (3)	0.034 (3)	0.018 (3)
C19	0.049 (2)	0.061 (3)	0.053 (2)	−0.0024 (19)	0.0113 (18)	0.0062 (19)
C22	0.0341 (17)	0.0364 (19)	0.047 (2)	−0.0009 (15)	−0.0045 (16)	0.0022 (16)
C23	0.0345 (18)	0.043 (2)	0.049 (2)	−0.0022 (16)	0.0005 (16)	−0.0013 (17)
O6	0.092 (2)	0.113 (3)	0.087 (2)	0.0019 (19)	0.0443 (19)	−0.0154 (18)
O7	0.0616 (16)	0.0548 (15)	0.0624 (16)	0.0000 (13)	0.0142 (13)	−0.0130 (13)
C24	0.131 (4)	0.061 (3)	0.069 (3)	−0.013 (3)	0.026 (3)	0.007 (2)
C25	0.075 (3)	0.066 (3)	0.064 (3)	−0.016 (2)	0.013 (2)	0.002 (2)
C26	0.048 (2)	0.053 (2)	0.054 (2)	0.0040 (18)	0.0093 (19)	0.0022 (18)
C27	0.052 (2)	0.114 (4)	0.065 (3)	−0.005 (2)	0.005 (2)	0.028 (3)
C28	0.053 (3)	0.168 (5)	0.064 (3)	0.011 (3)	0.005 (2)	0.042 (3)
C29	0.117 (4)	0.094 (4)	0.056 (3)	0.032 (3)	0.006 (3)	0.016 (3)
C31	0.0362 (18)	0.042 (2)	0.045 (2)	−0.0027 (15)	−0.0015 (16)	0.0017 (16)
C32	0.0402 (19)	0.044 (2)	0.052 (2)	0.0089 (16)	−0.0090 (17)	−0.0114 (18)
C33	0.053 (2)	0.068 (3)	0.074 (3)	0.014 (2)	−0.001 (2)	−0.020 (2)
C34	0.055 (3)	0.097 (4)	0.090 (3)	0.023 (3)	−0.003 (2)	−0.036 (3)
C35	0.065 (3)	0.064 (3)	0.118 (4)	0.019 (3)	−0.014 (3)	−0.031 (3)
C36	0.072 (3)	0.041 (2)	0.092 (3)	0.010 (2)	−0.026 (2)	−0.003 (2)
C37	0.059 (2)	0.034 (2)	0.063 (2)	0.0024 (18)	−0.021 (2)	0.0012 (19)
C38	0.052 (2)	0.049 (2)	0.054 (2)	−0.0086 (19)	−0.0105 (19)	0.0079 (19)
C39	0.080 (3)	0.052 (2)	0.073 (3)	−0.014 (2)	−0.020 (2)	0.014 (2)
C40	0.087 (3)	0.084 (4)	0.077 (3)	−0.027 (3)	0.016 (3)	0.022 (3)
C41	0.089 (3)	0.084 (3)	0.057 (3)	−0.024 (3)	0.014 (2)	0.011 (2)
C42	0.058 (2)	0.060 (3)	0.046 (2)	−0.010 (2)	0.0021 (19)	0.0021 (19)
C43	0.067 (3)	0.084 (3)	0.049 (2)	−0.006 (3)	0.004 (2)	−0.006 (2)
C45	0.0432 (19)	0.0332 (19)	0.041 (2)	0.0001 (16)	−0.0059 (16)	0.0038 (15)
C46	0.049 (2)	0.042 (2)	0.038 (2)	−0.0033 (17)	−0.0086 (16)	0.0032 (16)
O8	0.0635 (16)	0.0380 (14)	0.095 (2)	−0.0049 (13)	0.0196 (15)	0.0039 (14)
O1	0.0393 (14)	0.0691 (17)	0.0689 (17)	−0.0031 (12)	0.0090 (12)	0.0105 (13)
C44	0.039 (2)	0.051 (2)	0.051 (2)	0.0000 (18)	−0.0030 (17)	−0.0033 (18)
O3	0.0591 (15)	0.0508 (15)	0.0677 (17)	0.0008 (13)	0.0113 (14)	−0.0130 (13)
O4	0.0642 (17)	0.0409 (14)	0.104 (2)	−0.0070 (13)	0.0282 (16)	0.0030 (14)
O5	0.0396 (14)	0.0793 (18)	0.0827 (19)	−0.0116 (13)	0.0027 (13)	0.0030 (15)
O2	0.108 (2)	0.100 (2)	0.097 (2)	0.0240 (19)	0.056 (2)	−0.0096 (19)
C21	0.0404 (19)	0.045 (2)	0.055 (2)	−0.0018 (17)	−0.0005 (17)	−0.0070 (19)
C30	0.037 (2)	0.051 (2)	0.063 (2)	0.0050 (17)	0.0063 (18)	−0.0063 (19)
C20	0.064 (3)	0.069 (3)	0.057 (3)	0.009 (2)	0.007 (2)	−0.007 (2)

### Geometric parameters (Å, °)

**Table:**

C1—C6	1.367 (6)	C24—C29	1.381 (6)
C1—C2	1.370 (6)	C24—H24A	0.9303
C1—H1A	0.9289	C25—C26	1.390 (5)
C2—C3	1.371 (5)	C25—H25A	0.9298
C2—H2A	0.9297	C26—C27	1.376 (5)
C3—C4	1.384 (4)	C26—C30	1.492 (5)
C3—H3A	0.9303	C27—C28	1.379 (5)
C4—C5	1.382 (5)	C27—H27A	0.9293
C4—C7	1.486 (4)	C28—C29	1.360 (6)
C5—C6	1.379 (5)	C28—H28A	0.9297
C5—H5A	0.9310	C29—H29A	0.9294
C6—H6A	0.9303	C31—C45	1.366 (4)
C7—O1	1.223 (3)	C31—C32	1.439 (4)
C7—C8	1.509 (4)	C31—C30	1.504 (4)
C8—C22	1.360 (4)	C32—C33	1.399 (4)
C8—C9	1.437 (4)	C32—C37	1.419 (5)
C9—C10	1.399 (4)	C33—C34	1.372 (5)
C9—C14	1.422 (4)	C33—H33A	0.9308
C10—C11	1.375 (5)	C34—C35	1.381 (6)
C10—H10A	0.9293	C34—H34A	0.9305
C11—C12	1.360 (5)	C35—C36	1.351 (6)
C11—H11A	0.9298	C35—H35A	0.9303
C12—C13	1.361 (5)	C36—C37	1.417 (5)
C12—H12A	0.9309	C36—H36A	0.9308
C13—C14	1.408 (4)	C37—C38	1.442 (5)
C13—H13A	0.9293	C38—C46	1.400 (5)
C14—C15	1.436 (4)	C38—C39	1.422 (5)
C15—C23	1.405 (4)	C39—C40	1.364 (5)
C15—C16	1.423 (4)	C39—H39A	0.9308
C16—C17	1.354 (5)	C40—C41	1.358 (5)
C16—H16A	0.9299	C40—H40A	0.9306
C17—C18	1.378 (5)	C41—C42	1.372 (5)
C17—H17A	0.9292	C41—H41A	0.9312
C18—C19	1.371 (5)	C42—C46	1.406 (4)
C18—H18A	0.9556	C42—C43	1.450 (5)
C19—C23	1.402 (4)	C45—C46	1.433 (4)
C19—C20	1.455 (5)	C45—C44	1.466 (4)
C22—C23	1.430 (4)	O8—C44	1.197 (4)
C22—C21	1.471 (4)	O3—C20	1.370 (4)
O6—C43	1.210 (4)	O3—C21	1.380 (4)
O7—C44	1.376 (4)	O4—C21	1.189 (4)
O7—C43	1.397 (4)	O5—C30	1.226 (3)
C24—C25	1.376 (5)	O2—C20	1.214 (4)
			
C6—C1—C2	120.9 (4)	C27—C26—C25	119.2 (3)
C6—C1—H1A	119.6	C27—C26—C30	122.1 (3)
C2—C1—H1A	119.5	C25—C26—C30	118.7 (3)
C1—C2—C3	119.4 (4)	C26—C27—C28	120.3 (4)
C1—C2—H2A	120.3	C26—C27—H27A	119.9
C3—C2—H2A	120.3	C28—C27—H27A	119.8
C2—C3—C4	120.6 (4)	C29—C28—C27	120.3 (4)
C2—C3—H3A	119.7	C29—C28—H28A	119.9
C4—C3—H3A	119.6	C27—C28—H28A	119.7
C5—C4—C3	119.3 (3)	C28—C29—C24	120.2 (4)
C5—C4—C7	121.8 (3)	C28—C29—H29A	119.9
C3—C4—C7	119.0 (3)	C24—C29—H29A	119.9
C6—C5—C4	119.8 (4)	C45—C31—C32	119.8 (3)
C6—C5—H5A	120.1	C45—C31—C30	122.7 (3)
C4—C5—H5A	120.1	C32—C31—C30	117.5 (3)
C1—C6—C5	120.0 (4)	C33—C32—C37	119.4 (3)
C1—C6—H6A	120.0	C33—C32—C31	121.1 (3)
C5—C6—H6A	120.1	C37—C32—C31	119.5 (3)
O1—C7—C4	122.3 (3)	C34—C33—C32	120.7 (4)
O1—C7—C8	119.4 (3)	C34—C33—H33A	119.7
C4—C7—C8	118.2 (3)	C32—C33—H33A	119.6
C22—C8—C9	120.1 (3)	C33—C34—C35	120.0 (4)
C22—C8—C7	121.7 (3)	C33—C34—H34A	120.1
C9—C8—C7	118.3 (3)	C35—C34—H34A	119.9
C10—C9—C14	119.2 (3)	C36—C35—C34	121.0 (4)
C10—C9—C8	121.0 (3)	C36—C35—H35A	119.5
C14—C9—C8	119.7 (3)	C34—C35—H35A	119.5
C11—C10—C9	120.5 (4)	C35—C36—C37	121.3 (4)
C11—C10—H10A	119.8	C35—C36—H36A	119.4
C9—C10—H10A	119.7	C37—C36—H36A	119.4
C12—C11—C10	120.6 (4)	C36—C37—C32	117.6 (4)
C12—C11—H11A	119.6	C36—C37—C38	122.4 (4)
C10—C11—H11A	119.8	C32—C37—C38	120.0 (3)
C11—C12—C13	120.7 (4)	C46—C38—C39	117.4 (4)
C11—C12—H12A	119.6	C46—C38—C37	119.0 (3)
C13—C12—H12A	119.7	C39—C38—C37	123.6 (4)
C12—C13—C14	121.6 (4)	C40—C39—C38	121.1 (4)
C12—C13—H13A	119.3	C40—C39—H39A	119.4
C14—C13—H13A	119.1	C38—C39—H39A	119.5
C13—C14—C9	117.4 (3)	C41—C40—C39	120.8 (4)
C13—C14—C15	123.1 (3)	C41—C40—H40A	119.6
C9—C14—C15	119.5 (3)	C39—C40—H40A	119.6
C23—C15—C16	117.1 (3)	C40—C41—C42	120.7 (4)
C23—C15—C14	119.2 (3)	C40—C41—H41A	119.6
C16—C15—C14	123.7 (3)	C42—C41—H41A	119.7
C17—C16—C15	121.3 (4)	C41—C42—C46	120.1 (4)
C17—C16—H16A	119.4	C41—C42—C43	119.2 (4)
C15—C16—H16A	119.3	C46—C42—C43	120.6 (3)
C16—C17—C18	121.1 (4)	O6—C43—O7	115.7 (4)
C16—C17—H17A	119.5	O6—C43—C42	126.7 (4)
C18—C17—H17A	119.5	O7—C43—C42	117.5 (3)
C19—C18—C17	119.9 (4)	C31—C45—C46	121.5 (3)
C19—C18—H18A	115.6	C31—C45—C44	119.8 (3)
C17—C18—H18A	124.4	C46—C45—C44	118.7 (3)
C18—C19—C23	120.3 (4)	C38—C46—C42	119.9 (3)
C18—C19—C20	119.0 (4)	C38—C46—C45	120.1 (3)
C23—C19—C20	120.7 (3)	C42—C46—C45	120.0 (3)
C8—C22—C23	121.2 (3)	O8—C44—O7	116.1 (3)
C8—C22—C21	119.9 (3)	O8—C44—C45	125.1 (3)
C23—C22—C21	118.9 (3)	O7—C44—C45	118.7 (3)
C19—C23—C15	120.3 (3)	C20—O3—C21	123.4 (3)
C19—C23—C22	119.4 (3)	O4—C21—O3	115.9 (3)
C15—C23—C22	120.2 (3)	O4—C21—C22	125.8 (3)
C44—O7—C43	123.3 (3)	O3—C21—C22	118.3 (3)
C25—C24—C29	119.8 (4)	O5—C30—C26	120.9 (3)
C25—C24—H24A	120.1	O5—C30—C31	120.1 (3)
C29—C24—H24A	120.1	C26—C30—C31	118.7 (3)
C24—C25—C26	120.1 (4)	O2—C20—O3	116.7 (4)
C24—C25—H25A	119.9	O2—C20—C19	125.2 (4)
C26—C25—H25A	120.0	O3—C20—C19	118.1 (3)
